# Identifying individuals with physician-diagnosed chronic obstructive pulmonary disease in primary care electronic medical records: a retrospective chart abstraction study

**DOI:** 10.1038/s41533-017-0035-9

**Published:** 2017-05-15

**Authors:** Theresa M. Lee, Karen Tu, Laura L. Wing, Andrea S. Gershon

**Affiliations:** 1 Institute of Health Policy, Management and Evaluation, University of Toronto, Dalla Lana School of Public Health, 155 College Street, Suite 425, Toronto, ON M5T 3M6 Canada; 2 Institute for Clinical Evaluative Sciences, 2075 Bayview Avenue, G1 06, Toronto, ON M4N 3M5 Canada; 3 Department of Family and Community Medicine, University of Toronto, 500 University Ave, 5th Floor, Toronto, ON M5G 1V7 Canada; 4 Toronto Western Hospital Family Health Team-University Health Network, 399 Bathurst Street, Toronto, ON M5T 2S8 Canada; 5 Department of Medicine, University of Toronto, 200 Elizabeth Street, Suite RFE 3-805, Toronto, ON M5S 2C4 Canada; 6 Sunnybrook Health Sciences Centre, 2075 Bayview Ave, Toronto, M4N 3M5 ON Canada

## Abstract

Little is known about using electronic medical records to identify patients with chronic obstructive pulmonary disease to improve quality of care. Our objective was to develop electronic medical record algorithms that can accurately identify patients with obstructive pulmonary disease. A retrospective chart abstraction study was conducted on data from the Electronic Medical Record Administrative data Linked Database (EMRALD^®^) housed at the Institute for Clinical Evaluative Sciences. Abstracted charts provided the reference standard based on available physician-diagnoses, chronic obstructive pulmonary disease-specific medications, smoking history and pulmonary function testing. Chronic obstructive pulmonary disease electronic medical record algorithms using combinations of terminology in the cumulative patient profile (CPP; problem list/past medical history), physician billing codes (chronic bronchitis/emphysema/other chronic obstructive pulmonary disease), and prescriptions, were tested against the reference standard. Sensitivity, specificity, and positive/negative predictive values (PPV/NPV) were calculated. There were 364 patients with chronic obstructive pulmonary disease identified in a 5889 randomly sampled cohort aged ≥ 35 years (prevalence = 6.2%). The electronic medical record algorithm consisting of ≥ 3 physician billing codes for chronic obstructive pulmonary disease per year; documentation in the CPP; tiotropium prescription; or ipratropium (or its formulations) prescription and a chronic obstructive pulmonary disease billing code had sensitivity of 76.9% (95% CI:72.2–81.2), specificity of 99.7% (99.5–99.8), PPV of 93.6% (90.3–96.1), and NPV of 98.5% (98.1–98.8). Electronic medical record algorithms can accurately identify patients with chronic obstructive pulmonary disease in primary care records. They can be used to enable further studies in practice patterns and chronic obstructive pulmonary disease management in primary care.

## Chronic lung disease: Novel algorithm search technique

Researchers develop an algorithm that can accurately search through electronic health records to find patients with chronic lung disease. Mining population-wide data for information on patients diagnosed and treated with chronic obstructive pulmonary disease (COPD) in primary care could help inform future healthcare and spending practices. Theresa Lee at the University of Toronto, Canada, and colleagues used an algorithm to search electronic medical records and identify patients with COPD from doctors’ notes, prescriptions and symptom histories. They carefully adjusted the algorithm to improve sensitivity and predictive value by adding details such as specific medications, physician codes related to COPD, and different combinations of terminology in doctors’ notes. The team accurately identified 364 patients with COPD in a randomly-selected cohort of 5889 people. Their results suggest opportunities for broader, informative studies of COPD in wider populations.

## Introduction

Chronic obstructive pulmonary disease (COPD) is characterized by persistent airflow limitation and an enhanced chronic inflammatory airway response to noxious particles or gases such as tobacco smoke.^1^ COPD is one of the leading causes of death worldwide,^2–5^ with an estimated global prevalence of 64 million.^2^ Studies project an increase in morbidity and mortality from COPD due to the aging demographic and the delayed effects of previous increases in smoking rates.^6^ Despite its growing burden, COPD often remains incorrectly or under-diagnosed.^7, 8^ Primary care providers can play an important role in improving the management of patients with COPD. However, there is still a limited availability of population-wide data that can be used to build strategies for improvement of care, research and healthcare planning.

Previous work identifying people with and evaluating the burden of COPD have primarily been based on cross-sectional survey data and population cohorts.^5^ Self-reported measures for COPD in surveys have been validated against clinical records and physician diagnosis with relatively high accuracy, but are limited in clinical information.^9–12^ While population cohorts have been derived from health care claims from some administrative databases (particularly for populations with comprehensive health and drug coverage),^13^ they are limited in the depth and details of patient clinical information because they are created to manage financial transactions rather than for research purposes or patient care.^14^

Electronic medical record (EMR) systems are a potential comprehensive source of information on the processes and outcomes of patient care. EMRs include documentation of clinical encounters that occur within the physician office, including the patient medical history, laboratory test results, prescriptions, specialist consultation letters, discharge summaries, and diagnostic tests. The increasing use of EMRs in primary care settings provides a source of detailed clinical information that is not readily available in survey data or administrative databases, and is being used to study COPD among populations in the UK,^15, 16^ Sweden,^17^ Canada,^18–20^ and cross-nationally.^21–23^ The objective of this study was to determine whether patients with COPD could be accurately identified using the data contained in an EMR within Ontario, Canada.

## Results

### Reference standard

The abstracted cohort consisted of 364 patients with COPD out of a total of 5889 patients, resulting in a prevalence of physician-diagnosed COPD of 6.2%. Compared to people in the reference cohort, those with COPD were older and had a higher proportion of males. They were also more likely to have smoking history recorded in their charts (70% compared to 61% in those without COPD), and to have documented pulmonary function test (PFT) results (40% vs. 5% in patients without COPD; see Table 1). Review of the charts of patients with COPD who were non-smokers revealed seven patients who were subjected to long-term second-hand smoke and five patients with a history of occupational or environmental exposure.

**Table 1:** Study cohort characteristics by COPD diagnosis derived from primary care electronic medical record chart abstraction

	Total (*n* = 5889)	Patients without COPD (*n* = 5525)	Patients with COPD (*n* = 364)
Mean age, years (SD)	56.3 (±13.5)	55.4 (±13.2)	68.6 (±11.5)
Age>65 years, *n* (%)	1467 (24.9)	1244 (22.5)	223 (61.2)
Female, *n* (%)	3319 (56.4)	3157 (57.1)	162 (44.5)
Smoking history recorded, *n* (%)	3599 (61.1)	3345 (60.5)	254 (69.8)
Current smoker	656 (18.2)	554 (16.6)	102 (40.2)
Previous smoker	1121 (31.1)	994 (29.7)	127 (50.0)
Non-smoker (including second-hand smoke and environmental/occupational exposure)	1822 (50.6)	1797 (53.7)	25 (9.8)
Not recorded	2290 (38.9)	2180 (39.5)	110 (30.2)
Pulmonary Function Test record in EMR, *n* (%)	430 (7.3)	283 (5.1)	147 (40.4)

*COPD* chronic obstructive pulmonary disease, *SD* standard deviation

### EMR algorithm validation

The algorithms tested for identifying patients with COPD in the EMR all had high specificity and negative predictive value (NPV), but varied in their sensitivity and positive predictive value (PPV) (see Table 2). An algorithm consisting of documentation in the cumulative patient profile (CPP) alone had a PPV of 95%, but detected only slightly over half (56%) of the patients with COPD from the reference standard.

**Table 2:** Test characteristics of various electronic medical record COPD algorithms when validated against an abstracted patient chart reference standard (*n* = 5889, COPD prevalence = 6.2%)

Algorithm	True positive (*n*)	True negative (*n*)	False positive (*n*)	False negative (*n*)	Sensitivity (95% CI)	Specificity (95% CI)	Positive predictive value (95% CI)	Negative predictive value (95% CI)	Positive likelihood ratio (95% CI)	Negative likelihood ratio (95% CI)	Diagnostic odds ratio (95% CI)
Cumulative patient profile in the EMR											
Problem list & past medical history	205	5514	11	159	56.3% (51.1–61.5)	99.8% (99.6–99.9)	94.9% (91.1–97.4)	97.2% (96.7–97.6)	282.9 (155.7–514.0)	0.44 (0.39–0.49)	646.3 (345.3–1209.6)
Physician billing codes for COPD (any of ‘chronic bronchitis’ [491], ‘emphysema’ [492] or ‘other COPD’ [496])											
≥1 billing code (ever)	188	5405	120	176	51.6% (46.4–56.9)	97.8% (97.4–98.2)	61.0% (55.3–66.5)	96.8% (96.4–97.3)	23.8 (19.4–29.1)	0.49 (0.44–0.55)	48.1 (36.6–63.3)
≥2 billing codes in 1 year	100	5510	15	264	27.5% (22.9–32.4)	99.7% (99.6–99.8)	87.0% (79.4–92.5)	95.4% (94.9–96.0)	101.2 (59.4–172.3)	0.73 (0.68–0.77)	139.1 (79.8–242.8)
Positive smoking history											
Current smoker	102	4971	554	262	28.0% (23.5–32.9)	90.0% (89.2–90.8)	15.5% (12.9–18.6)	95.0% (94.4–95.6)	2.8 (2.3–3.4)	0.80 (0.75–0.85)	3.5 (2.7–4.5)
Ex-smoker	118	4531	994	246	32.4% (27.6–37.5)	82.0% (81.0–83.0)	10.6% (8.9–12.6)	94.9% (94.2–95.5)	1.8 (1.5–2.1)	0.82 (0.76–0.89)	2.2 (1.7–2.8)
Medication prescriptions in the EMR											
Tiotropium or ipratropium (or ipratropium/ salbutamol)	198	5508	17	166	52.2% (46.9–51.4)	99.9% (99.8–100.0)	97.9% (94.8–99.4)	96.9% (96.5–97.4)	176.8 (109.0–286.8)	0.46 (0.41–0.52)	386.5 (230.0–649.3)
Tiotropium	186	5524	11	159	51.1% (45.8–56.3)	100.0% (99.9–100.0)	99.5% (97.1–100.0)	96.9% (96.4–97.3)	271.3 (149.1–493.5)	0.46 (0.41–0.51)	587.5 (313.4–1101.1)
Ipratropium or ipratropium/salbutamol	47	5509	16	317	12.9% (9.6–16.8)	99.7% (99.5–99.8)	74.6% (62.1–84.7)	94.6% (93.9–95.1)	44.6 (25.5–77.8)	0.87 (0.84–0.91)	51.1 (28.6–91.0)
Combinations of cumulative patient profile, prescription and billing code algorithms											
CPP ***OR*** Tiotropium ***OR*** (ipratropium ***AND***≥1 billing code) ***OR***≥3 billing codes in 1 year	280	5506	19	84	76.9% (72.2–81.2)	99.7% (99.5–99.8)	93.6% (90.3–96.1)	98.5% (98.1–98.8)	223.7 (142.3–351.6)	0.23 (0.19–0.28)	966.0 (578.8–1612.1)
CPP ***OR*** Any COPD prescription ***OR***≥2 billing codes in 1 year	288	5483	42	76	79.1% (74.6–83.2)	99.2% (99.0–99.5)	87.3% (83.2–90.7)	98.6% (98.3–98.9)	104.1 (76.7–141.3)	0.21 (0.17–0.26)	494.7 (333.3–734.4)
Combinations of cumulative patient profile, prescription, billing code algorithms, and positive smoking history (current or ex-smoker)											
CPP ***OR*** Tiotropium ***OR*** (ipratropium ***AND***≥1 billing code) ***OR***≥3 billing codes in 1 year ***OR*** positive smoking history	325	3969	1556	39	89.3% (85.6–92.3)	71.8% (70.6–73.0)	17.3 % (15.6–19.1)	99.0 % (98.7–99.3)	3.2 (3.0–3.4)	0.15 (0.11–0.20)	21.3 (15.2–29.8)
CPP ***OR*** Any COPD prescription ***OR***≥2 billing codes in 1 year ***OR*** positive smoking history	329	3955	1570	35	90.4% (86.9–93.2)	71.6% (70.4–72.8)	17.3% (15.6–19.1)	99.1% (98.8–99.4)	3.2 (3.0–3.4)	0.13 (0.10–0.18)	23.7 (16.6–33.7)

*CI* confidence interval, *COPD* chronic obstructive pulmonary disease, *EMR* electronic medical record, *SD* standard deviation

Algorithms using at least one COPD billing code (any of 491, 492 or 496) captured only about half of the patients with COPD and had a PPV of 61%. Algorithms that searched for at least two of any of the billing codes in the span of 1 year had a lower sensitivity of 28%, but a higher PPV at 87%. When looking at COPD medications, we found varying degrees of accuracy with tiotropium and ipratropium (or combinations). Algorithms searching for ipratropium (or ipratropium/salbutamol) prescriptions had a sensitivity of 13% and PPV of 75%, while searching for prescriptions of tiotropium resulted in a sensitivity of 51% and an excellent PPV of 99.5%. Combining all prescriptions increased the sensitivity slightly to 52% and had a PPV of 98%. A recorded history of being a current smoker or ex-smoker captured 28 and 30% of patients with COPD, respectively. However, using smoking history alone resulted in a very low PPV of 16 and 11% respectively.

Algorithms using a combination of different EMR components (CPP, prescriptions, billing codes) had higher sensitivity than the individual components alone, while maintaining high scores for PPV, specificity and NPV. Our final algorithm optimizing PPV and sensitivity included COPD documentation in the CPP; a prescription for tiotropium at any time; or a prescription for ipratropium (or ipratropium/salbutamol) in conjunction with a COPD billing code at anytime in the chart; or at least 3 COPD billing codes within 1 year. This algorithm resulted in 77% sensitivity and PPV of 94%, with the highest diagnostic odds ratio (DOR) of 966, high positive likelihood ratio (LR+) of 224 and modest negative likelihood ratio (LR−) of 0.23.

An alternative algorithm could increase the sensitivity marginally by searching for COPD documentation in the CPP; any of the COPD-specific prescriptions; or at least 2 COPD physician billing codes within 1 year. This algorithm had a sensitivity of 79%, PPV of 87%, a very high DOR of 495, LR+ of 104 and LR− of 0.21, resulting in prevalence of 5.6% in the population compared to the 6.2% in the reference cohort.

Including a positive smoking history to either of the two optimized algorithms increased the sensitivity to a maximum of 90%, but resulted in over 25% reduction in specificity and 75% reduction in and PPV.

### Discordance analysis

Using the search algorithm that optimized PPV and sensitivity, there were 84 false negatives and 19 false positives. Of the 84 false negatives less than six (< 8%) patients were not correctly identified with COPD because their primary care CPP phrasing met exclusion rules. Specifically, there was a query “?” or “possible” label next to the diagnosis of COPD in the CPP despite a definitive diagnosis in other parts of the patient record. Approximately a quarter of the patients had less than three COPD billing codes from their family physicians. Fifty-eight (65%) were not identified because of what appeared to be an incomplete primary care CPP, where there was no mention of COPD in the CPP despite a diagnosis and documentation in the body of the chart or in consultation notes from other physicians. All 85 patients did not have a prescription in their family physician’s EMR for tiotropium, ipratropium or ipratropium/salbutamol. Out of the 19 false positives, 11 (58%) had COPD listed in the CPP as a possible diagnosis, followed by text not considered by our exclusion rules (e.g., “Asthma/COPD”), or it appeared that the CPPs were not updated as there was evidence in the chart that the diagnosis was only suspected or had been ruled out. Eight (42%) met the billing code criteria but had no further documentation in the charts indicative of COPD.

## Discussion

We conducted a validation study that confirmed that an EMR algorithm could accurately identify patients with physician-diagnosed COPD using the data components contained in primary care EMRs. Our final algorithm optimizing PPV and sensitivity searched for indication of COPD in the CPP; prescription for tiotropium at any time; prescription for ipratropium (or ipratropium/salbutamol) in conjunction with a COPD billing code; or at least 3 COPD physician-billing codes within 1 year. This validated algorithm could be used to accurately identify a cohort of patients with COPD in primary care to conduct future studies in COPD quality of care, clinical audit, prediction modeling, and health care utilization patterns.

When compared to previously described COPD EMR algorithms from other jurisdictions,^15, 18–20^ our algorithm performed with the highest PPV published to date. A high PPV indicates a high proportion of positive results that are true positives, which is useful in identifying cohorts that actually have COPD. As seen in previous studies, there is a trade-off between accuracy and capture rate when selecting an algorithm. For instance, Cooke et al.^24^ described a COPD algorithm using administrative data with a high sensitivity of over 90% with lower PPV of 58%. With a different set of input variables in their model, this shifted to a moderate sensitivity of 71.9%, and improved PPV of 71.2%.^24^ Similarly, using EMR data, Kadhim-Saleh et al.^19^ had algorithm results ranging from a lower sensitivity of 41% and higher PPV of 80%, to a higher sensitivity of 82.1% and lower PPV of 72.1%.^19^ As these components often counterbalance each other, users of algorithms should evaluate the trade-offs and purpose of the algorithm they choose to apply in their research. Additional considerations include the implication of errors (e.g., false positives and false negatives), and the prevalence of the disease in the population (if it is rare or common). Sensitivity should be optimized in cases where it is important to minimize false negatives and detect as many cases as possible (e.g., disease surveillance, high risk associated with missed detection, public health education or preventive/early detection, and intervention purposes). It was important for our algorithm to achieve the highest PPV possible in order minimize the number of false positives in identifying future cohorts of patients that are correctly identified as having COPD. In doing so, it would improve the accuracy of the algorithm and ensure any analysis related to COPD in our database would reflect care specific to the disease of interest as much as possible. High accuracy of the algorithm was also indicated by the high DOR, which measures the algorithm’s effectiveness, and high LR+, which assesses the performance of the algorithm in finding positive results.

There have been two other algorithms in the literature that use the EMR data to identify patients with COPD. One algorithm (using the case definition of “obstructive chronic bronchitis” (491.2), “emphysema” (492), or “chronic airway obstruction” (496) in the billing history or in the problem list; or tiotropium, ipratropium, or salbutamol and other drugs for obstructive airway disease listed under medication; with the exclusion of people under the age of 35 years and those who fulfill only the medication criteria alone and also have asthma) that was applied in different clinics and regions across Canada,^18–20^ showed varying sensitivity (41–82%), specificity (92–99%), PPV (37–80%), and NPV (88–98%) for the identification of COPD across sites. These varying results suggest that further studies are needed to understand how to best use EMR algorithms for diverse populations. An algorithm used in the UK^15^ had access to a different set of EMR data components and codes than those used in this study, including those for spirometry or PFTs and was therefore not comparable to our study. Although PFT results could not be included in our algorithm, it would be possible to incorporate billing codes for spirometry by linking the EMR data with Ontario’s administrative data set in future studies.

We found that looking in the CPP alone or COPD billing codes alone yielded sub optimal sensitivity (<60%). This suggests that the documentation and billing patterns for COPD within primary care physicians in their EMRs is variable and/or incomplete. Searching for COPD prescriptions alone in the algorithm also had low sensitivity as not all patients with COPD are given prescriptions for tiotropium, ipratropium, or combination of ipratropium/salbutamol by their family physician. Patients may have received other medications such as short-acting bronchodilators, but as these medications are not specific for COPD and are also given for other conditions (e.g., asthma or acute bronchitis) they were excluded from our algorithm.^20^ Additionally, medication prescribed by specialists may be missing in the primary care EMR as the accuracy and completeness of the medication list in the EMR is dependent on individual family physicians to record them.

Our study shows that searching for billing codes alone or COPD medications alone identifies patients with COPD with a lower degree of accuracy than also searching the free text in the CPP. However, there are also challenges associated with distinguishing COPD from other respiratory conditions such as acute bronchitis or asthma.^25–27^ These complexities are reflected in notations within the EMR entries and CPP (e.g., “?Asthma/COPD”[sic] in the CPP and problem list indicating possible but not ruled-out diagnosis of COPD), making it challenging to use automated text searching algorithms to identify the most up-to-date and relevant information. The inaccuracies recorded in the CPP highlight the need to improve recording of COPD diagnosis among primary care practice within EMRs.

Searching for COPD-specific medications to identify patients with COPD also presented some challenges. In a previous study by Coleman et al.,^20^ inclusion of COPD medication (e.g., salbutamol) in their algorithm resulted in nearly half of the results being a misdiagnosis, as the medications included could also be used for acute bronchitis, chronic cough, or asthma.^20^ In consultation with respirologists, we reduced the list to three medications that were as specific as possible to COPD to limit the number of false positives. However, we still noted that tiotropium and ipratropium may both be used for severe cases or exacerbations of asthma.^28, 29^ There were also instances where patients were provided with samples or trials of drugs of tiotropium without a confirmed diagnosis of COPD to see whether presenting symptoms improved. These resulted in a small number of false-positive misclassifications. Furthermore, as medications are manually entered to the EMR and physicians may or may not utilize medication drop down lists, we may not have accounted for all misspellings and short-forms of the drug names, or prescriptions provided by other providers and specialists outside the primary care practice.

We did not include smoking history in our final chosen algorithms. Smoking remains a significant risk factor for COPD^1^ and patients’ smoking history can be captured in the EMR. As seen in Table 1, we found a higher proportion of EMR documented smoking history among patients with COPD compared to the whole study cohort (70 vs. 61%). These rates of documentation are higher than a previous Swedish study where one-third of patients had information on smoking recorded in their records.^17^ However, while smoking history could be helpful in supporting a positive diagnosis clinically and could increase the sensitivity, it was not included as a data component in the EMR algorithms due to the low PPV and lack of precision. When smoking status (current smoker or ex-smoker) was added as a factor in our top algorithms, the sensitivity rose > 90%, but the specificity decreased to 72% and PPV to only 17% (see Table 2). This result is due to the fact that smoking status was not captured for everyone and a positive smoking history is not solely diagnostic of COPD.

PFTs are widely available and have been recommended for confirmation of COPD diagnosis.^1^ However, we found that PFT results were recorded in the EMR for only 40% of the patients with COPD and 5% of patients without COPD, consistent with previous studies.^25, 27^ These low rates of PFTs could be because the PFT performed and the COPD diagnosis predates the start of the EMR record, or because a PFT was not done. With linkage to the administrative data, it is possible to differentiate these possibilities and can be performed in future studies. In addition, PFTs that are performed outside of the clinic are often sent in via fax or scanned in, thus the results are not always captured in a text searchable format in the EMR. The limited availability and inconsistent formatting of the results did not allow for us to include PFT results in our EMR algorithm. These findings are similar to those found in Sweden, where only 29% of the primary healthcare centres had extractable PFT data due to lack of common structure for its documentation.^17^ In a few cases where a computed tomography scan consistent with COPD was recorded, we accepted this as a definitive case of COPD. Our study highlights the need to standardize and automate the capture of diagnostic test results related to COPD.

Other considerations for this study include limitations to generalizability. Our study uses EMR data from primary care practice in a voluntary subset of the Ontario population using one type of EMR software within the study period. Despite this, patients in Electronic Medical Record Administrative data Linked Database (EMRALD®) have similar characteristics to the general population in terms of presence of chronic diseases and co-morbid conditions.^30^

The literature on developing COPD algorithms show that there may be bias for diagnosis according to patients’ sex, race, level of education, and level of severity of COPD resulting in under- or over-diagnosis.^31, 32^ These socioeconomic and demographic factors were not accounted for in this study, and we were unable to determine severity of COPD. While these issues are beyond the scope of this study, they would be important areas for future research and could be studied with a larger cohort of COPD patients and in conjunction with the administrative data.

## Conclusion

We conducted a validation study that confirmed that an EMR algorithm can accurately identify patients with physician-diagnosed COPD using data components contained in primary care EMRs. Our COPD cohort had characteristics consistent with those in the literature, suggesting good validity of our reference standard. Our findings indicate the importance of keeping the CPP up to date in primary care practice, which would improve the accuracy of EMR algorithms to identify patients with COPD. There is also a need to improve recording of diagnostic tests for COPD. Researchers and other users of the EMR data should take caution and note the limitations of using billing codes alone or medication lists alone to identify patients with COPD.

As EMRs become increasingly used across jurisdictions, it presents many opportunities to study detailed clinical information on a broad population with COPD, including nationally and internationally.^17, 21–23^ This study shows that primary care EMR data can be a promising source of data to study populations in the community with COPD in Ontario, the most populous province in Canada. Using EMR algorithms to identify patients with COPD has the potential to help study quality of care, appropriate use of pharmacological therapy, patient outcomes, health care utilization patterns, and clinical and economic consequences with the ultimate goal of improving patient care and outcomes.

## Methods

We conducted a validation study using retrospective chart abstraction to identify a reference cohort of individuals with physician-diagnosed COPD. This cohort was used as a reference standard to test a variety of EMR algorithms to identify patients with COPD. This study was approved by the institutional review board at Sunnybrook Health Sciences Centre, Toronto, Canada.

### Data source

EMRALD® held at the Institute for Clinical Evaluative Sciences (ICES) was used as the data source to create the reference standard.^33^ At the time of study, EMRALD^®^ provided a sampling frame of 73,014 adult patients aged 20 years or older as of 31 December 2010, and included all patient chart data entered in the EMR from 1986 to 2011. Patients in EMRALD^®^ have been found to provide a good representation of the Ontario population.^30^ Data are collected on a semi-annual basis. The inclusion criteria for patients were: to have a valid date of birth; a valid health insurance number; and have made at least one visit to any of the 83 participating physicians in the year preceding EMR data abstraction from the clinics. The physicians had to have used the EMR for at least 2 years so as to optimize the completeness of data.^30^ These data sets were linked using unique encoded identifiers and analyzed at ICES.

A random sample of 5889 patients aged 35 years and over was taken from the sampling frame using Structured Query Language (Microsoft SQL Server [2008]). Three trained chart abstractors performed manual chart reviews on all available patient charts to determine whether patients had a diagnosis of COPD, classifying each encounter with the patient as indicating “definite COPD” (i.e., diagnosis by the physician), “possible COPD” (i.e., a prescription for a short-acting bronchodilator that could indicate an airway disease, but not necessarily for COPD), “COPD ruled out” (i.e., a negative test result or ruling out by the physician), or “no mention of COPD”. Abstractors assessed the cumulative patient profile, each entry in the chart, which included diagnostic information such as PFT results and prescriptions for COPD-related medications including ipratropium, combined ipratropium and salbutamol, and tiotropium. Inter- and intra-rater reliabilities of the chart abstractions were verified by double-abstraction of 10% of the charts and calculating kappa-scores. The study team re-reviewed patients’ charts that were marked as “possible” or as “definite” but had no COPD prescriptions in the medication field to verify the accuracy of the abstraction.

### EMR algorithm development

The patients identified as “definite COPD” after the chart abstraction review were used as the reference standard against which various EMR algorithms identifying patients with COPD were tested. Algorithms were developed from searching within EMR data components for terminology specific to COPD, including its acronyms, full spelling, and common misspellings. The CPP algorithm searched for evidence of terms that implied positive COPD diagnosis in the CPP (i.e., problem list and past medical history). The prescription algorithm searched the medication list of the EMR for COPD-specific medications including their generic and trade names within varying time intervals and whether they were prescribed at any point in time vs. being currently active prescriptions. Algorithms for billing codes searched for physician billing codes for COPD (“chronic bronchitis” (491), “emphysema” (492), or “other COPD” (496) within varying time frames. Finally, a search for the smoking status of the patient (current smoker, ex-smoker, non-smoker, unspecified) was determined by the most recent smoking history section of the cumulative patient profile.

### Analysis

Algorithm performance was analyzed using the concepts of diagnostic test evaluation using the manual chart abstraction as the reference standard. We calculated the sensitivity, specificity, PPV, NPV, 95% confidence intervals (CI) (determined by using an exact method based on a binomial distribution), and prevalence of COPD for each of the algorithms using Microsoft SQL. All algorithms developed from individual EMR components (CPP, prescriptions, physician billings) were compared to assess how they impacted the sensitivity, specificity, PPV and NPV scores. Each algorithm’s DOR LR+, and LR− were calculated for further assessment. Different variations of EMR components were combined to maximize each of the scores.

### Code availability

The computational and statistical codes used for analysis are available from the corresponding author on request.

### Data availability

The data set used in this study is held securely in coded format at the ICES. Although the data sharing agreements prohibit ICES from making the dataset publicly available, access may be granted to those who meet the conditions for confidential access.
